# Phenocopy syndrome of behavioral variant frontotemporal dementia: a systematic review

**DOI:** 10.1186/s13195-019-0483-2

**Published:** 2019-04-01

**Authors:** Elizabeth Sakamoto Valente, Paulo Caramelli, Leandro Boson Gambogi, Luciano Inácio Mariano, Henrique Cerqueira Guimarães, Antônio Lúcio Teixeira, Leonardo Cruz de Souza

**Affiliations:** 1 Programa de Pós-Graduação em Neurociências, Universidade Federal de Minas Gerais (UFMG), Instituto de Ciências Biológicas (sala 100, Bloco M1), Avenida Presidente Antônio Carlos, 6627 – Pampulha, Belo Horizonte, MG 31270-010 Brazil; 2 Departamento de Clínica Médica, Faculdade de Medicina, Universidade Federal de Minas Gerais, Av. Professor Alfredo Balena, 190/sl 243, Belo Horizonte, MG 30130-100 Brazil; 3 Neuropsychiatry Program, Department of Psychiatry and Behavioral Sciences, McGovern Medical School, The University of Texas Health Science Center at Houston (UTHealth), 1941 East Road, Houston, TX 77054 USA

**Keywords:** Frontotemporal dementia, Phenocopy, *C9orf72*

## Abstract

**Background:**

The phenocopy syndrome of behavioral variant of frontotemporal dementia (phFTD) refers to patients presenting with neuropsychiatric symptoms mimicking the behavioral variant frontotemporal dementia (bvFTD), but lacking frontotemporal atrophy/hypometabolism on neuroimaging and not evolving to dementia during the follow-up. It is important to recognize phFTD for clinical and research purposes.

**Objective:**

The aim of this study was to perform a systematic review of the available literature on phFTD taking into account its clinical, cognitive, imaging, genetic, and pathological features.

**Methods and results:**

We searched for the following terms in two electronic databases (PubMed and Scopus): “frontotemporal dementia and slowly progressive,” “frontotemporal dementia and phenocopy,” “frontotemporal dementia and non-progressive,” “frontotemporal dementia and benign progression,” and “frontotemporal dementia and benign.” We did not include review articles. Papers had to be written in English, French, Portuguese, or Spanish. Overall, 235 studies were retrieved in the initial search. A total of 31 studies composed the final selection, comprising 292 patients. Patients with phFTD are predominantly male and have no major cognitive deficits, with globally preserved executive functions and episodic memory. Some cases (*n* = 7) of slowly progressive FTD have been associated with *C9orf72* genetic expansion. There are only four reports of phFTD neuropathological data, with two patients with no neurodegenerative findings and two with frontotemporal lobar degeneration with ubiquitin-positive inclusions.

**Conclusion:**

The neurobiological underpinnings of phFTD remain unknown. It is controversial whether phFTD belongs to the FTD spectrum. Studies with biomarkers and pathological data are needed to solve the phFTD conundrum.

## Background

Frontotemporal dementia (FTD) is a neurodegenerative disorder characterized by progressive deterioration of behavior and/or language associated with marked atrophy of frontal and/or temporal lobes [1]. FTD is the second most frequent cause of early-onset dementia, also affecting older subjects [1]. FTD comprises three distinct clinical phenotypes: behavioral variant, semantic variant of primary progressive aphasia (PPA), and non-fluent/agrammatic PPA. The behavioral variant of FTD (bvFTD) is the most frequent subtype [1].

Patients with bvFTD have progressive changes in personality and social conduct. According to consensual diagnostic criteria for bvFTD [2], the diagnosis of possible bvFTD requires at least three of six characteristics: disinhibition, apathy/inertia, loss of empathy and/or sympathy, perseveration/compulsive behaviors, hiperorality, and neuropsychological profile of executive dysfunction with relative sparing of episodic memory and visuospatial skills. Diagnosis of probable bvFTD additionally requires functional impairment and prominent signs of focal frontotemporal involvement in either structural or functional neuroimaging exams [2]. Definite bvFTD is reserved for patients with known pathogenic genetic mutation or with histopathological evidence of frontotemporal lobar degeneration (FTLD) [2].

The estimated mean survival for patients with bvFTD ranges from 6 to 8 years since symptom onset [3]. However, over the past few years, some studies have identified a group of patients clinically indistinguishable from typical FTD who does not progress to frank dementia on follow-up. These patients fulfill criteria for possible bvFTD and have limited or no imaging abnormalities, such as focal prefrontal atrophy on magnetic resonance imaging (MRI) or frontal hypometabolism on fluorodeoxyglucose-positron emission tomography (FDG-PET) [4]. Since their condition remains stable over many years, such group has been called “phenocopy” of FTD (phFTD), FTD “phenocopy syndrome,” “non-progressive” FTD, “benign” FTD, or slowly progressive FTD [4–7]. It is worth emphasizing that patients with phFTD do not satisfy the diagnostic criteria for probable bvFTD since neuroimaging is unremarkable or almost normal.

Despite efforts to characterize phFTD, results have been controversial. For instance, while some studies reported preserved global cognitive efficiency in phFTD [8–10], others described that phFTD patients perform worse than controls in general measures of cognition [5, 11].

From a neuropathological point of view, the question also remains unclear as there are only a few histopathological studies of these patients. As some patients with slowly progressive bvFTD have been diagnosed with *chromosome 9 open reading frame 72* (*C9orf72*) mutation [6, 12], it has been suggested that phFTD could be due to FTLD, the so-called indolent variant of FTD [13]. The hypothesis of an “indolent” FTD places the phenocopy group in the FTD spectrum and raises the issue whether phFTD represents a slow neurodegenerative process. Conversely, phFTD has been conceptualized as late-onset forms of psychiatric disorders including late-onset bipolar disorder and personality disorders [8, 14, 15].

In practical terms, phFTD has been regarded as a clinical entity similar to bvFTD but with relatively normal cognitive performance, intact activities of daily living, no neuroimaging features of bvFTD, and without clinical progression over three or more years of follow-up [7]. However, several questions remain unanswered. How is it possible to explain the behavioral overlap between phFTD and typical bvFTD in the absence of structural or functional brain abnormalities in the former? Are there any other clinical and cognitive features distinguishing phFTD from bvFTD? The identification of phFTD is highly relevant for clinical purposes in order to provide good clinical care, family support and to establish realistic outcomes for these patients. The aim of this study was to perform a systematic review of the available literature about phFTD taking into account its clinical, cognitive, imaging, biological, genetic, and pathological aspects.

## Methods

We conducted a systematic review according to the guidelines proposed by the Preferred Reporting Items for Systematic Reviews and Meta-Analyses (PRISMA) [16]. This search was independently performed by two investigators (ESV and LCS) in July 2018. We searched for the following terms in two electronic databases (PubMed and Scopus): “frontotemporal dementia and slowly progressive,” “frontotemporal dementia and phenocopy,” “frontotemporal dementia and non-progressive,” “frontotemporal dementia and benign progression,” and “frontotemporal dementia and benign.” We adopted the following filters: clinical articles, comparative studies, historical articles, journal articles, letter, classical articles, case report, comments, and clinical trials. We did not include review articles or abstracts of scientific meetings. They had to be written in English, French, Portuguese, or Spanish. No chronological limits were adopted. Disagreement of eligibility (*n* = 1) was resolved through a consensual agreement between authors (ESV and LCS).

We carried out the following procedure: (1) titles and abstracts were screened and non-pertinent studies were excluded; (2) after this initial screen, selected articles were subsequently read in full-text and non-pertinent ones were excluded.

This systematic review was registered in the PROSPERO international platform under the number CRD42018107060.

## Results

**Fig. 1:**
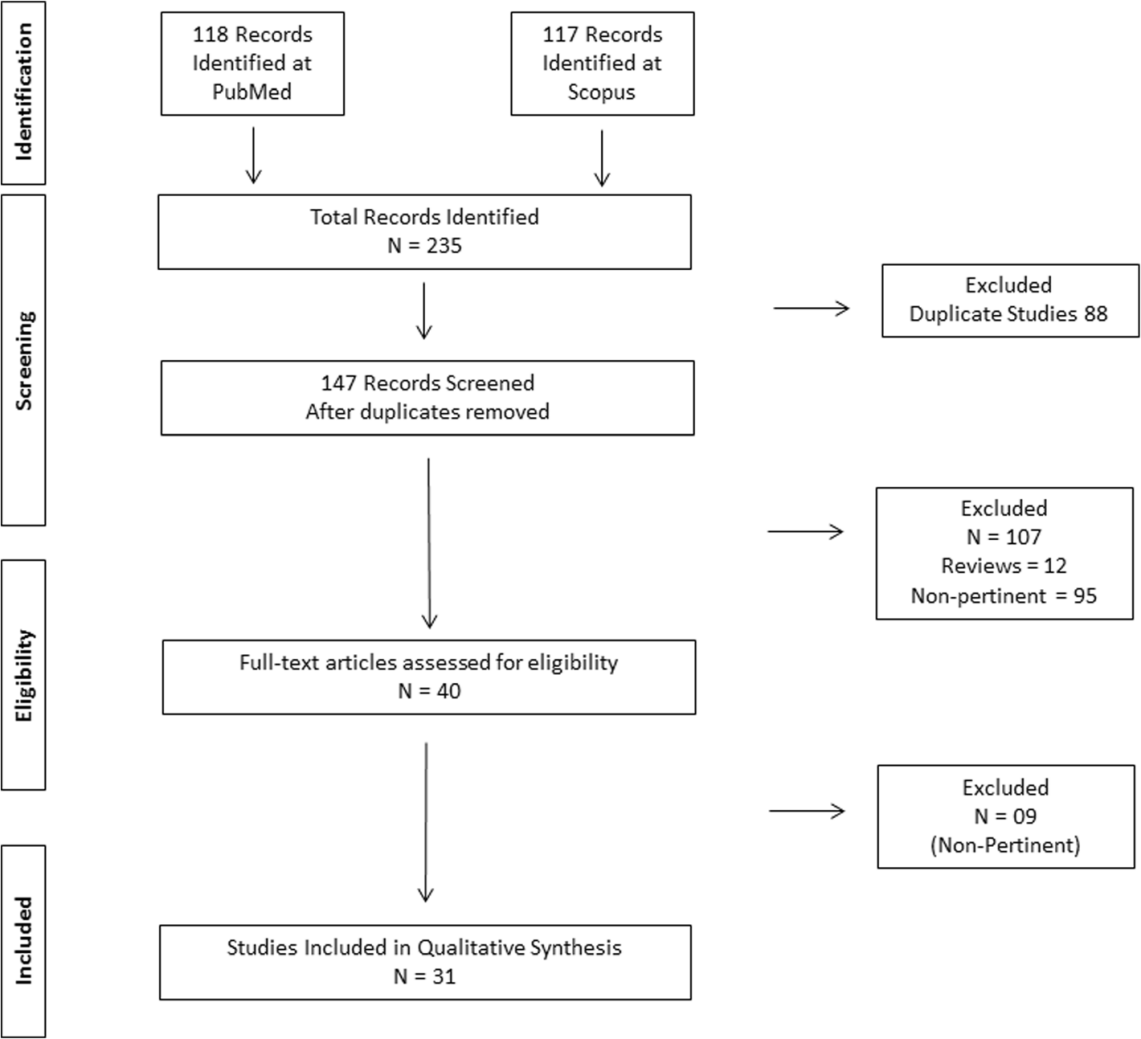
PRISMA flow diagram for studies of phenocopy syndrome of frontotemporal dementia

A total of 235 studies were retrieved in the initial search. A total of 31 studies composed the final selection (see Fig. 1), comprising 292 patients. Table 1 presents the main findings from the selected studies. Table 1 also provides detailed information on how each study defined phFTD.

**Table 1:** Synthesis of studies included in the present review

Title	Authors	Year	Population	Design and methods	Results
Progression in Frontotemporal Dementia: identifying a benign behavioral variant by magnetic resonance imaging	Davies et al.	2006	*N* = 31 patients with bvFTD bvFTD was defined by Neary et al. (1998) and McKhan et al. criteria (2001).	Longitudinal study (follow-up of 15 years). The study compared the prognosis of two groups: (1) bvFTD with normal or borderline MRI (2) bvFTD with marked frontotemporal atrophy Good prognosis was defined as independent for ADL after 3 years of follow-up, while poor prognosis was defined as death or institutionalization within the same period.	The groups with normal or borderline MRI (*N* = 16 patients) had better prognosis (longer survival to institutionalization or death) than those (*N* = 15 patients) with marked frontotemporal atrophy.
Behavioural variant Frontotemporal Dementia: Not all it seems?	Kipps et al.	2007	*N* = Two patients with bvFTD according to Neary et al. (1998) criteria	Case report with clinical, neuropsychological and neuroimaging (MRI and FDG-PET).	Case 1 had a clinical decline over 10 years from symptom onset, progressive atrophy, and frontotemporal hypometabolism. Case 2 did not develop atrophy or hypometabolism and remained clinically stable, a decade from symptom onset.
Novel cerebrospinal fluid biomarkers of axonal degeneration in frontotemporal dementia	Mattsson et al.	2008	*N* = 24 FTD patients according to Neary et al. (1998) criteria, including: (1) 13 patients with rapidly progressive FTD (defined by marked brain atrophy after 3 years of follow-up) (2) 11 patients with slowly progressive FTD (defined by normal or inconclusive MRI, but abnormal SPECT/PET after at least 5 years of follow-up)	This cross-sectional study compared two groups of FTD patients on CSF peptide analysis (mass spectrometry).	Rapidly progressive FTD group presented a higher level of neurofilament light chain protein.
Executive function in progressive and nonprogressive behavioral variant frontotemporal dementia	Hornberger et al.	2008	*N* = 90 participants, including: (1) 27 patients with progressive bvFTD (2) 23 patients with non-progressive bvFTD (3) 40 controls FTD patients fulfilled Neary et al. (1998) criteria. Non-progressive patients had (1) absence of marked frontotemporal atrophy and no decline on ACE-R over a 3-year period.	This retrospective cohort study compared neuropsychological and behavioral tests across two bvFTD groups.	The non-progressors performed in the normal range on executive tasks. Progressors were impaired on Digit Span Backward, Hayling Test, Letter Fluency, and Trails B. A subgroup of progressors had normal executive functioning.
Can progressive and non-progressive behavioural variant frontotemporal dementia be distinguished at presentation?	Hornberger et al.	2009	*N* = 71 patients with bvFTD according to Neary et al. (1998) criteria, including: (1) Progressive (*N* = 45) (2) Non-progressive bvFTD patients (*N* = 26) Progression was defined as cognitive (measured on ACE-R) and functional decline over 3 years of follow-up.	This retrospective cohort study (1991–2007) compared behavioral (CBI) and cognitive data between groups.	Progressors had worse performance on the ACE-R, worse functional profile and higher frequency of distractibility, stereotypic speech. Depression was more frequent in non-progressors.
Combined magnetic resonance imaging and positron emission tomography brain imaging in behavioural variant frontotemporal degeneration: refining the clinical phenotype	Kipps et al.	2009	24 bvFTD patients according to Brun et al. (1994) and Neary et al. (1998) criteria, including: (1) bvFTD with abnormal MRI (*N* = 15) (2) bvFTD with normal MRI (*N* = 9) Twelve healthy controls were also included.	This cross-sectional study compared cognitive and behavioral measures (MMSE, ACE, CDR, CBI); and FDG-PET metabolism between bvFTD subgroups	Most of bvFTD with abnormal MRI (14/15) showed frontotemporal hypometabolism on FDG-PET and clinical progression. Most bvFTD patients with normal MRI (7/9) had no hypometabolism and no clinical progression. Almost a third of bvFTD with abnormal MRI had ACE-R scores overlapping with the normal MRI subgroup.
Determinants of survival in behavioral variant frontotemporal dementia	Garcin et al.	2009	*N* = 91 patients diagnosed with bvFTD, according to Neary et al. (1998) criteria, including: (1) Phenocopies (*N* = 24) defined as a lack of progression on the ACE and ADLs over a period of 3 years follow-up and normal MRI at presentation 2) “Pathological” bvFTD (*N* = 67)	This retrospective review compared neurological and psychiatric assessments, cognitive and behavioral measures (CBI, MMSE, and ACE) and neuroimaging (MRI) data between subgroups.	Phenocopy cases were younger and had longer survival than “pathologic” FTD.
Activities of daily living in behavioral variant frontotemporal dementia: differences in caregiver and performance-based assessments	Mioshi et al.	2009	*N* = 18 bvFTD patients fulfilling Neary et al. (1998) criteria, including: (1) Phenocopy (*N* = 10), defined as bvFTD patients without evidence of atrophy on MRI at presentation and with no progression over at least 2 years of follow-up (2) Pathologic bvFTD (*N* = 8)	This cross-sectional study compared subgroups on behavioral, neuropsychological measures and also on functional scales of activities of daily living (DAD and AMPS) and a qualitative assessment).	PhFTD and pathologic bvFTD did not differ on the DAD, but differed at AMPS and qualitative rating assessment.
Rate of Change of Functional Abilities in Frontotemporal Dementia	Mioshi et al.	2009	*N* = 26 participants, including: (1) bvFTD (*N* = 5 patients fulfilling Neary et al. criteria) (2) bvFTD phenocopies = 10 patients, with no atrophy in their initial MRI scans as well as on follow-up scans after 12 months phenocopies) fulfilling Neary et al. criteria (3) Semantic variant of PPA (*N* = 8) (4) PNFA (*N* = 3)	This prospective cohort study compared behavioral and neuropsychological tests (initiation, planning, and execution scores) and functional scales of daily living between groups.	Only phenocopies did not show a significant functional decline after 12 months. The decline in ADL and cognitive scores were significantly correlated.
How preserved is episodic memory in behavioral variant frontotemporal dementia?	Hornberger et al.	2010	*N* = 62 participants, including: (1) bvFTD patients: progressors (*N* = 39) (2) bvFTD phenocopies (*N* = 23) (3) AD patients (*N* = 64) (4) Healthy controls (*N* = 64) bvFTD patients (progressors and phenocopies) fulfilled Neary et al. criteria. bvFTD cases were classified retrospectively into progressive and phenocopy based on the presence/absence of change over a 3-year period on ACE and ADLs.	This retrospective cross-sectional compared groups on behavioral (CBI) and neuropsychological tests, including episodic memory test (RAVLT).	Episodic memory deficits in “progressive” bvFTD were similar to AD. bvFTD phenocopies performed better than bvFTD progressors and AD patients.
Survival in a German Population with Frontotemporal Lobar Degeneration	Nunnemann et al.	2011	*N* = 124 FTLD patients, including: (1) bvFTD (*N* = 81) (2) Semantic variant of PPA (*N* = 21) (3) PNFA (*N* = 22) The diagnosis of FTLD was established according to Neary et al. criteria (1998)	Prospective follow-up study. Clinical data and interviews with caregivers	Phenocopy cases were not identified in this sample.
Neural Correlates of Episodic Memory in Behavioral Variant Frontotemporal Dementia	Pennington et al.	2011	*N* = 59 participants, including: (1) “classical” bvFTD (*N* = 14) (2) bvFTD phenocopies (*N* = 6) (3) AD (*N* = 14) (4) Healthy controls (*N* = 15) Both “classical” bvFTD and phenocopies fulfilled Rascovsky criteria (2011). Phenocopy was defined by relatively stable, non-progressive behavioral disturbance with a lack of progression over 2 years or more and preserved activities of daily living.	Cross-sectional study. Cognitive assessment and MRI scanning with ratings of regional brain atrophy.	BvFTD and AD patients were similarly impaired on memory scores. bvFTD phenocopies did not differ from controls on memory scores and atrophy ratings.
The neuropsychological correlates of pathological lying: evidence from behavioral variant frontotemporal dementia	Poletti et al.	2011	*N* = 1 patient with clinical features of bvFTD according to Neary’s criteria (1998)	Case report. Neurological, neuropsychiatric, neuropsychological, and neuroimaging exam.	Pathological lying was observed in a patient 57-year-old, with suggestive bvFTD and lack of prefrontal hypometabolism.
Atypical, slowly progressive behavioural variant frontotemporal dementia associated with *c9orf72* hexanucleotide expansion	Khan et al.	2012	*N* = 384 patients (either with FTD or AD) were screened for *C9orf72* mutation. 87 had bvFTD and 23 of them had *C9orf72* mutation. Four patients (2 C9+, 2 C9-) were identified as slowly progressive course (bvFTD-SP) and were selected. These patients fulfilled Rascovsky criteria for bvFTD, had normal or limited atrophy on brain MRI and had no decline on CDR over at least 2 years after initial assessment.	Case reports presenting neuropsychological and functional tests; structural MRI/with voxel based-morphometry (VBM) analysis.	Both C9+ bvFTD-SP patients initially met the criteria for possible bvFTD and remained stable on neuropsychological and functional measures during the follow-up.
Differential Impairment of Source Memory in progressive Versus Non-progressive behavioral Variant Frontotemporal Dementia	Irish et al.	2012	*N* = 35 participants, including: (1) progressive bvFTD (*N* = 7 patients) (2) non-progressive bvFTD (*N* = 12) (3) Healthy controls (*N* = 16). The diagnosis of bvFTD was established according to Neary et al. (1998) criteria. bvFTD patients were classified retrospectively into progressive versus non-progressive. Definition of non-progressors was based on the absence of decline on the ACE-R over the 3-year period following diagnosis.	Cross-sectional study. Cognitive tests including a *source monitoring task.*	Progressive bvFTD patients had more severe impairment of temporal source memory than non-progressive bvFTD patients.
Phenocopy or variant: a longitudinal study of very slowly progressive frontotemporal dementia	Brodtman et al.	2013	*N* = 2 patients	Case report of a patient and his father with very slowly progressive cognitive decline and personality change. Neuropsychological tests, MRI, and PET were performed on patient. Genetic testing in both cases. Histopathological examination was available for the patient’s father.	PET and MRI scans were unchanged over 15 years in the patient. Neuropsychological assessments revealed no cognitive deterioration over this period. Histopathology of his father demonstrated early-stage frontotemporal lobar degeneration with ubiquitin.
Tracking the progression of social cognition in neurodegenerative disorders	Kumfor et al.	2014	*N* = 61 participants including: (1) bvFTD (*N* = 20 patients) (2) AD (*N* = 17 patients) (3) Healthy controls (*N* = 24) bvFTD were classified on two subgroups according to brain atrophy at baseline: *N* = 8 bvFTD with limited brain atrophy [bvFTD-la] and *N* = 12 bvFTD with marked atrophy [bvFTD-ma]. The diagnosis of bvFTD was established according to Rascovsky et al. (2011) criteria. The clinical follow-up was inferior to 3 years.	Prospective study, with behavioral and neuropsychological tests, including social cognition tests (Ekman 60 and TASIT).	BvFTD with and without brain atrophy were impaired on the general cognition and emotion recognition tasks. On the sarcasm detection task, only the bvFTD-ma group was impaired. On the emotion recognition and sarcasm tasks, the bvFTD-ma group declined more rapidly than bvFTD-la and AD patients. The bvFTD-la group remained stable over time on the emotion recognition and sarcasm measures.
Two Distinct Amnesic Profiles in Behavioral variant Frontotemporal Dementia	Bertoux et al.	2014	*N* = 134 participants including: (1) 56 AD (*N* = 56) (2) bvFTD (*N* = 44) (3) Phenocopies (N = 12) (4) Controls (*N* = 22) The diagnosis of bvFTD was established according to Rascovsky et al. (2011) criteria. Phenocopies fulfilled criteria for possible bvFTD and had no change on cognitive and functional measures over a 3-year period.	Cross-sectional study, with neuropsychological assessment, focused on episodic memory (the Free and Cued Selective Reminding Test (FCSRT)).	A subgroup of bvFTD had episodic memory impairment, similar to AD. Phenocopies and non-amnestic FTD performed similar to controls on the FCSRT.
The Added Value of 18-Fluorodeoxyglucose-Positron Emission Tomography in the Diagnosis of the Behavioral variant of Frontotemporal Dementia	Kerklaan et al.	2014	*N* = 52 patients with suspected bvFTD according to Neary et al. (1998) criteria, and with no typical atrophy on brain MRI.	Retrospective cohort study, with neuropsychological and behavioral assessment, structural neuroimaging, and 18-FDG-PET. Patients with functional decline over 2 years of follow-up (bvFTD/fd+) were compared with patients without functional decline (bvFTD/fd−).	The sensitivity of FDG-PET for bvFTD/fd+ was 47% at a specificity of 92%. The 18F-FDG-PET was abnormal in only 1 of the 8 cases of the bvFTD/fd− group.
Familial benign frontotemporal deterioration with C9ORF72 hexanucleotide expansion	Gomés-Tortoza et al.	2014	*N* = 3 patients of the same family with benign FTLD associated with *C9orf72* gene hexanucleotide expansion	Case report of three patients from the affected family	Two siblings had cognitive complaints, preserved ADLs, mild-moderate atrophy on MRI and evolved to slow progression of deficits over more than 10 years. Their mother had mild cognitive impairment and slowly progressive dementia over a time frame of more than 30 years.
Altered network connectivity in frontotemporal dementia with *C9orf72* hexanucleotide repeat expansion	Lee et al.	2014	*N* = 42 participants, including: (1) 28 patients with bvFTD (14 *C9orf72* mutation carriers and 14 non carriers) (2) 14 healthy controls bvFTD patients met Neary et al. (1998) and Rascovsky et al. (2011) criteria.	Cross-sectional study with neuropsychological exam and functional MRI (task-free).	Compared to controls and mutation carriers, *C9orf72* (−) bvFTD patients had increased DMN connectivity. *C9orf72* (+) bvFTD patients with early stage or slowly progressive symptoms (*N* = 4) had salience network disruption and DMN enhancement.
Slowly progressive frontotemporal lobar degeneration caused by the *C9ORF72* repeat expansion: a 20-year follow-up study	Suhonen et al.	2015	*N* = 1 patient with slowly progressive FTLD, with mixed features of semantic variant of PPA and bvFTD.	Case report, presenting neuropsychological data, MRI, and FDG-PET	52-year-old man with semantic deficits associated with *C9orf72* mutation. MRI revealed mild frontal atrophy whereas FDG-PET showed significant hypometabolism in the temporal and frontal lobes. The disease did not progress to dementia for over 20 years from the onset.
Progression in Behavioral Variant Frontotemporal Dementia: A Longitudinal Study	Devenney et al.	2015	*N* = 58 patients with bvFTD, according to Rascovsky et al. criteria (2011). At inclusion, 38 patients had probable bvFTD and 20 had possible bvFTD. The follow-up varied from 1 to 6 years.	This prospective cohort study assessed the prognostic value of clinical, genetic, neuropsychological, and neuroimaging parameters.	Nine out of 20 patients with possible bvFTD remained stable over time, while 11 progressed to probable bvFTD. Most of progressors (eight out 11) were *C9orf72* carriers. A family history of dementia, episodic memory deficits, and clinical findings (e.g., parkinsonism, frontal release signs) were key features of clinical progression.
Structural and functional brain abnormalities place phenocopy frontotemporal dementia (FTD) in the FTD spectrum	Steketee et al.	2016	*N* = 38 participants, including: (1) bvFTD patients (*N* = 11) (2) phFTD patients (*N* = 7) (3) Healthy controls (*N* = 20) The diagnosis of bvFTD was established according to Rascovsky et al. (2011) criteria. phFTD fulfilled criteria for possible bvFTD, had no imaging abnormalities suggestive of bvFTD, and no clinical decline for at least 1 year after initial diagnostic. None of the phenocopy patients had *C9orf72* mutation.	This cross-sectional study compared volumetric and perfusion MRI measures across groups.	Gray matter volume did not differ between phFTD and controls, whereas bvFTD showed extensive frontotemporal atrophy. Compared to FTD, phFTD patients had increased perfusion in the left prefrontal cortex.
Psychiatric diagnoses underlying the phenocopy syndrome of behavioural variant frontotemporal dementia	Gossink et al.	2016	*N* = 52 participants, including: (1) bvFTD patients (*N* = 19) (2) phFTD (*N* = 33) The diagnosis of bvFTD was established according to Rascovsky et al. (2011) criteria. Phenocopy cases were considered as possible bvFTD, with normal neuroimaging, without functional decline over at least 1 year of follow-up.	Retrospective chart review; neurological and psychiatric evaluation.	In the phenocopy group, 85.2% of patients had psychiatric or psychological conditions (e.g., cluster C personality traits) which were more frequent than in the bvFTD group (47.4%).
Slowly progressive behavioural presentation in two UK cases with the *R406W MAPT* mutation	Wood et al.	2016	*N* = 2 patients with slowly progressive personality changes, over more than 10 years.	Case reports, with clinical follow-up, neuropsychological test, and brain MRI	Both patients presented a slowly progressive behavioral disorder with predominantly right temporal lobe atrophy, associated with the *R406W MAPT* mutation.
The bvFTD phenocopy syndrome: a clinicopathological report	Devenney et al.	2016	*N* = 2 patients fulfilling criteria for possible bvFTD. The clinical follow-up for cases 1 and 2 was of 16 and 5 years, respectively.	Report of clinical and pathological findings in two patients with slowly progressive behavioral disorders.	Both cases showed behavioral changes consistent with bvFTD. They did not show brain atrophy or hypometabolism on neuroimaging. Both patients did not have FTLD at postmortem pathological exam.
Late life bipolar disorder evolving into frontotemporal dementia mimic	Dols et al.	2016	*N* = 4 patients with bipolar disorder type 1. All patients fulfilled Rascovsky criteria (2011) for possible bvFTD.	Report of cases with psychiatric, neurological, neuropsychological, and neuroimaging data.	All cases had early- and late-onset bipolar disorder, who subsequently developed gradually progressive behavioral and social-emotional changes. During the clinical follow-up (3 to 7 years), there was no progression to “probable” bvFTD.
Functional connectivity and microstructural white matter changes in phenocopy frontotemporal dementia	Meijboom et al.	2016	*N* = 36 participants, including: (1) bvFTD patients (*N* = 12) (2) phFTD patients (*N* = 7) (3) Healthy controls (*N* = 17) The diagnosis of bvFTD was established according to Rascovsky et al. criteria (2011). phFTD patients were defined by marked behavioral changes with no clinical decline during 1 year follow-up.	This cross-sectional study compared functional (DMN) and structural (DTI) connectivity across groups.	Compared to controls, phFTD had enhanced DMN connectivity and subtle microstructural changes in frontal tracts. Compared to phFTD, bvFTD had lower enhanced DMN connectivity and more extensive WM abnormalities on DTI analysis.
Slowly progressive behavioral frontotemporal dementia with *C9orf72* mutation. Case report and review of the literature	Llamas-Velasco et al.	2018	*N* = 1 patient with bvFTD according to Rascovsky criteria (2011)	Case report.	The patient had personality changes and functional decline over more of 30 years. She carried *C9orf72* hexanucleotide expansion.
The behavioural variant frontotemporal dementia phenocopy syndrome is a distinct entity – evidence from a longitudinal study	Devenney et al.	2018	*N* = 43 participants, including: (1) phFTD patients (*N* = 16) (2) Probable bvFTD patients (*n* = 27). The diagnosis of bvFTD was established according to Rascovsky et al. criteria (2011). phFTD patients had relative functional and cognitive preservation and no atrophy on brain MRI. The minimal follow-up was of 3 years.	This is a prospective cohort study, presenting behavioral (CBI) and neuropsychological tests. Genetic screening for the *C9orf72* was performed Long-term follow-up (13–21 years) were available in six phFTD cases.	Most of phFTD patients remained stable over time, including those with long follow-up (13–21 years). Only one of 16 phFTD cases (6.25%) had the *C9orf72* expansion.

*ACE-R* Addenbrooke’s Cognitive Examination-Revised, *ADL* activities of daily living, *AMPS* Assessment of Motor and Process Skills, *bvFTD* behavioral variant frontotemporal dementia, *CBI* Cambridge Behavioral Inventory, *CDR* Clinical Dementia Rating, *CSF* cerebrospinal fluid, *DAD* Disability Assessment of Dementia, *DMN* default mode network, *c9orf72* chromosome 9 open reading frame 72, *DTI* diffusion tensor imaging, *FCSRT* Free and Cued Selective Reminding Test, *FDG-PET* fluorodeoxyglucose-positron emission tomography, *FTD* frontotemporal dementia, *FTLD* frontotemporal lobar degeneration, *MRI* magnetic resonance imaging, *PET* positron emission tomography, *phFTD* phenocopy of FTD, *PPA* primary progressive aphasia, *PMID* PubMed Identifier Number, *RAVLT* Rey Auditory Verbal Learning Test, *SPECT* single-photon emission computed tomography, *TASIT* The Awareness of Social Inference Test

Results are presented in five parts: part I, epidemiological aspects; part II, cognitive and functional profiles; part III, behavioral and psychiatric profiles; part IV, neuroimaging; and part V, biomarkers, genetic and neuropathological findings in phFTD.

### Part I: Epidemiological aspects

The frequency of phFTD among bvFTD series was variable [3–5, 8, 17, 18], ranging from 0 [18] to 52% [4] in the selected studies. Most studies reported a higher percentage of men among phFTD patients [3–5, 8, 11, 17, 19–25]. Considering all the reported cases (*n* = 292), there is a clear male predominance (male = 255, female = 25, missing data = 12), with a male to female ratio of 10:1. Some studies reported that patients with phFTD were younger (45–65 years) than bvFTD (50–75 years) [3, 17], but this was not observed in other series [4, 5, 21, 22]. Non-progressive FTD usually does not present with neurological signs (e.g., primitive reflexes) on physical examination [20], and family history for dementia is typically absent [3, 20].

### Part II: Cognitive and functional profiles

Most studies did not find differences between phFTD patients and healthy controls on measures of global cognitive efficiency, such as the Mini-Mental State Examination (MMSE) and Addenbrooke’s Cognitive Examination-Revised (ACE-R) [10, 19, 22]. However, Steketee et al. reported that phFTD patients had significantly lower MMSE scores than controls [11]. Similarly, patients with non-progressive FTD performed worse than controls on ACE-R in another study [5].

Most studies reported that phFTD patients had better global cognitive performance than bvFTD, as measured by the MMSE and the ACE-R [3, 5, 19, 21].

One study assessed structural (MRI) and functional neuroimaging (FDG-PET) in a group of 24 patients with bvFTD [26]. bvFTD patients were classified according to brain MRI in a subgroup with atrophy pattern suggestive of bvFTD (*n* = 15) and a subgroup without abnormalities (*n* = 9). bvFTD patients with abnormal MRI performed worse than the bvFTD group with normal MRI on the ACE-R, but almost a third of bvFTD with abnormal MRI had ACE-R scores overlapping with the normal MRI subgroup [26]. This finding suggests that global cognitive efficiency may not be a good measure to differentiate progressive from non-progressive FTD (phFTD).

There is evidence indicating that episodic memory performance may distinguish progressive from non-progressive patients presenting with FTD-related behavioral disorders. Patients with phFTD syndrome had normal performance on episodic memory tests, performing better than typical bvFTD patients [10, 19, 27]. Moreover, memory scores seemed to be very sensitive to detect progressive bvFTD cases at initial presentation [20, 27]. Source memory tasks may also distinguish bvFTD from phFTD at initial presentation. Patients with progressive bvFTD had impairment on temporal and spatial source retrieval, while phFTD patients displayed only temporal source deficits [22].

Executive tasks did not seem to provide a distinction between bvFTD and phFTD, as some bvFTD patients had normal executive performance [5]. phFTD patients performed better than bvFTD in executive tests, such as Digit Span, Letter Fluency, Trail Making, and Hayling [5]. However, up to 20% of bvFTD patients had normal performance on these same tests [5]. Of note, the frequency of dysexecutive syndrome at presentation did not differ phFTD from bvFTD in a cohort of 91 subjects [3].

A longitudinal study investigated emotion recognition and sarcasm detection in bvFTD [28], suggesting that social cognition tasks may be a useful tool to differentiate progressive from non-progressive bvFTD. However, in this study, bvFTD patients had a functional decline at baseline and the follow-up was inferior to 3 years, preventing their classification as phFTD [28].

The functional profile of phFTD has also been investigated. One study compared performance on activities of daily living (ADL) in phFTD and bvFTD [25], according to two ADL measures: a caregiver-based scale, the Disability Assessment of Dementia (DAD), and a patient-based scale, the Assessment of Motor and Process Skills (AMPS). The minimal follow-up period for the phFTD group in this study was 5 years. There was no difference between phFTD and bvFTD in DAD scale, but there was a clear distinction on the performance-based measure (AMPS), with bvFTD patients exhibiting worse performance than phFTD [25]. Another study [24] evaluated the rate of change in ADLs in phFTD and bvFTD. Although both groups had similar levels of functional skills at baseline, bvFTD patients deteriorated in ADLs over 12 months, while phFTD patients did not. Taken together, these data support that there is no evidence of functional impairment in phFTD, suggesting that the assessment of daily activities may be useful to differentiate phFTD from bvFTD.

### Part III: Behavioral and psychiatric profiles

Some studies compared behavioral features between bvFTD and phFTD. One study [21] compared progressor and non-progressor patients regarding their profile on the Cambridge Behavioral Inventory (CBI) at initial presentation. There was no difference on bvFTD core diagnostic features between progressors and non-progressors. However, distractibility and stereotypic speech were more common in progressors, while current depression was more frequent in non-progressors [21].

Stereotypical and compulsive behaviors have also been associated with clinical and functional decline in a large series of patients followed up to 5 years [20]. Confabulation was reported in one phFTD patient [29]. On the contrary, other studies showed that bvFTD and phFTD were indistinguishable on behavioral features at presentation [4, 5, 25].

One retrospective study [17] investigated psychiatric and psychological features in patients with phFTD, reporting a higher frequency of recent life events, relationship problems, and cluster C personality traits in this group when compared with bvFTD patients. Bipolar disorder seemed to be more frequent in phFTD patients than in bvFTD group, and one phFTD patient was considered to have autism spectrum disorder [17].

Dols et al. reported four patients with bipolar disorder slowly developing a clinical syndrome marked by apathy, disinhibition, loss of empathy, stereotypical behavior, and compulsiveness, similar to bvFTD [14]. Patients had modest cognitive impairment and did not progress over 3 to 7 years of follow-up. Neuroimaging was unrevealing and *C9orf72* screening was negative in all cases. These authors hypothesized that end-stage bipolar disorder could be the underlying cause of the phenocopy syndrome in these patients [14].

In sum, only one study systematically assessed psychiatric antecedents among phFTD patients [17]. There is some evidence suggesting a clinical overlap between phFTD and bipolar disorder in the elderly.

### Part IV: Neuroimaging

Patients with phFTD do not exhibit evident frontotemporal atrophy in brain MRI or focal hypometabolism/hypoperfusion in functional neuroimaging methods. It is worth emphasizing that focal frontotemporal atrophy is a marker of clinical and functional decline during the follow-up, ruling out phFTD [4, 23].

Steketee et al. compared bvFTD, phFTD, and healthy controls with quantitative methods in functional and structural MRI [11]. The phFTD group (*n* = 7) showed cortical atrophy, most prominently in the right temporal lobe, whereas bvFTD group (*n* = 11) had extensive frontotemporal atrophy [11]. Compared to bvFTD and controls (*n* = 20), cerebral perfusion was increased in phFTD patients, with higher perfusion in the left prefrontal cortex [11].

Functional connectivity and white matter (WM) microstructure were investigated in bvFTD and phFTD [9]. Compared to controls (*n* = 17), phFTD patients (*n* = 7) showed higher connectivity on the default mode network (DMN) than bvFTD patients (*n* = 12). There were frontotemporal WM abnormalities in both bvFTD and phFTD groups, but they more pronounced in bvFTD [9]. Increased DMN connectivity was also reported in slowly progressive patients with *C9orf72* expansion and no characteristic atrophy on structural MRI [30].

Brain metabolism on FDG-PET may be abnormal in cases of normal brain MRI [23, 26]. A typical pattern of frontotemporal hypometabolism is usually associated with a functional decline over the years, but patients with clinical behavioral features of bvFTD and abnormal metabolism on FDG-PET may also remain stable over the years [23, 26, 31]. Normal MRI has a high negative predictive value of normal FDG-PET [26], while FDG-PET increases the sensitivity of the diagnosis of bvFTD [23]. As FDG-PET is a sensitive marker of neurodegeneration, the results showing the absence of typical frontotemporal metabolism in most phFTD patients reinforce the absence of an underlying neurodegenerative process in this condition. Taken together, while data with quantitative methods suggest that phFTD patients share some structural and functional abnormalities with bvFTD [9, 30], other findings are not indicative of an underlying neurodegenerative process [26].

Table 2 shows a synthesis of the comparison of clinical, cognitive, behavioral, and imaging features between phFTD and bvFTD.

**Table 2:** Comparison of behavioral variant frontotemporal dementia (bvFTD) and phenocopy syndrome of FTD (phFTD)

	bvFTD	phFTD
Sex	No sex predominance	Male predominance
Family history for dementia	Generally present	Rare
Behavioral symptoms	Frontal behavior	Frontal behavior
Global cognitive efficiency	Mild to severe impairment	Generally preserved
Executive function	Mild to severe impairment	Normal to mild impairment
Episodic memory	Moderate to severe impairment	Normal
Activities of daily living	Moderate to severe impairment	No impairment
MRI	Frontotemporal atrophy	Normal to minimal changes
FDG-PET	Frontotemporal hypometabolism	Usually normal

*MRI* magnetic resonance imaging, *FDG-PET* fluorodeoxyglucose-positron emission tomography

### Part V: Biomarkers, genetics, and neuropathological data

*C9orf72* expansion has been identified in patients with slowly progressive FTD [6, 12, 32, 33]. For instance, three cases of slowly progressive FTD associated with *C9orf72* expansion were reported in the same family [12].

The *R406W* microtubule-associated protein tau (*MAPT*) mutation is typically associated with a slowly progressive memory decline with symmetrical frontotemporal atrophy on MRI. A novel phenotype associated with the *R406W* mutation has been identified, marked by a slowly progressive behavioral disorder related to predominant right temporal lobe atrophy [34].

So far, only four patients with phFTD underwent autopsy [13, 35]. Two phFTD cases with behavioral disorders, mild dysexecutive function, and unchanged neuropsychological testing during follow-up (5 and 10 years) did not have FTLD pathology on postmortem pathological exam [35].

On the other hand, spongiosis and gliosis associated with ubiquitin-positive inclusions were reported in one patient featuring typical FTD behavioral symptoms, but no abnormalities on both structural and functional neuroimaging after 3 years of follow-up [36]. In the same study, the profile of peptides in the cerebrospinal fluid (CSF) differed between patients with rapidly progressive FTD (*n* = 13) and slowly progressive FTD (*n* = 11), indicating that these may be valuable markers of establishing FTD prognosis [36].

FTLD with ubiquitin pathology was also found in a patient with a 20-year history of behavioral disorders with slow functional decline [13]. Staining for *fused-in-sarcoma* (FUS) and *TAR DNA-binding protein 43* (TDP-43) proteins was negative, and no amyloid plaques were observed; tau pathology was scarce [13].

## Discussion

For many years, bvFTD has been considered a clinically homogeneous condition marked by stereotypical behaviors, typical neuropsychological profile (severe executive dysfunction and relative sparing of episodic memory), and shorter survival than Alzheimer’s disease. Recent data from longitudinal studies with bvFTD patients with cognitive, molecular, and neuroimaging tools have challenged this classic clinical profile, highlighting the phenotypical heterogeneity of FTD. More specifically, a subgroup of slowly progressive patients with no evident neuroimaging features of FTD has been recognized. FTD patients with no or slow decline over at least 3 years after symptom onset have been referred as phenocopies of bvFTD (phFTD). In other terms, phFTD is characterized by changes in behavior but with normal neuroimaging, thus fulfilling criteria for possible bvFTD. Moreover, phFTD patients have no or very slow cognitive and functional decline on follow-up.

The phenocopy syndrome of FTD is a clinical and scientific challenge. From a clinical perspective, distinguishing bvFTD from phFTD is crucial for prognosis purpose, clinical care as well as for patient and family counseling and support. From a scientific perspective, the inclusion of phFTD patients in cohorts of bvFTD patients may hinder the development of disease-modifying strategies against FTD. Researchers in the field of FTD should be aware of phFTD for optimal cognitive and behavioral characterization of these patients.

There is a variable frequency of phFTD patients among FTD series [18, 20]. Methodological issues, such as different diagnostic definitions of phFTD and distinct periods of follow-up, hamper establishing a precise prevalence of phFTD.

There is some evidence that cognitive measures may help to differentiate bvFTD from phFTD. Some studies found that bvFTD and phFTD differ in terms of performance in episodic memory tests [10, 19, 27]. Consensual diagnostic criteria for bvFTD state that episodic memory is relatively spared in bvFTD [2]. However, there is increasing evidence that episodic memory impairment occurs in bvFTD [37, 38] in a similar degree as observed in Alzheimer’s disease [19, 27, 39–41]. Actually, amnesia in bvFTD is associated with the involvement of medial temporal structures, such as hippocampal and perihippocampal regions [37, 39–41]. phFTD patients seem to have normal performance on episodic memory tests, suggesting preservation of the Papez’s circuit. These findings suggest that episodic memory impairment may be a marker of progressive FTD, distinguishing bvFTD from phFTD [19, 21, 27].

Executive functions seem to be more impaired in bvFTD than in phFTD [5, 21], but dysexecutive syndrome at presentation does not seem to be a prognostic factor for bvFTD patients [3]. Moreover, a subset of bvFTD patients may not manifest prominent executive dysfunction at presentation, performing within normal values in executive tests [5, 42, 43]. Therefore, the absence of executive dysfunction in a patient with behavioral features of bvFTD should not be considered as a marker of non-progression. A word of caution is needed here as studies in the field did not always include healthy controls, therefore limiting the interpretation of the prognostic value of cognitive parameters.

The overall preservation of cognitive functions including episodic memory, executive functions and social cognition in phFTD is in line with the lack of significant neuroimaging abnormalities in these patients. By definition, phFTD patients do not exhibit clear frontotemporal involvement in brain imaging. However, a recent study reported functional connectivity changes and microstructural WM abnormalities in phFTD [9]. Compared to phFTD, patients with bvFTD had a similar topographical pattern of alterations, but with more intense abnormalities [9]. Compared to healthy controls, patients with phFTD had a mild increase in DMN connectivity, while bvFTD had a lower increase in the same measure. These authors proposed that DMN increased connectivity would be a compensatory mechanism to early impairment in neuronal functioning [9]. They also suggested that those findings supported the hypothesis that phFTD may belong to the FTD spectrum or might constitute a prodromal phase of bvFTD [9]. More studies are necessary to test this hypothesis.

A fundamental question is whether there is an underlying neurodegenerative process in phFTD. There are only four phFTD reports with postmortem neuropathological assessment. No FTLD pathology was found in two cases [35], while FTLD pathology was documented in two patients [13, 36].

Neurodegeneration biomarkers could shed some light into the question whether phFTD belongs to FTD spectrum. To the best of our knowledge, there is no study investigating phFTD patients with molecular neuroimaging markers such as flortaucipir, which has already been used in FTD patients [44, 45]. So far, there is only one study investigating CSF markers in phFTD patients [36]. In the next future, CSF biomarkers and/or molecular neuroimaging with pathophysiological markers may contribute to the in vivo distinction between phFTD and bvFTD.

To further complicate the scenario, recent studies have reported slowly progressive bvFTD in carriers of *C9orf72* expansion [6, 12, 20, 32, 33]. The *C9orf72* mutation has important diagnostic implications. The presence of pathogenic mutation in bvFTD patients, regardless of neuroimaging findings, establishes the diagnosis of “definite” bvFTD [2]. One half of *C9orf72* carriers initially met criteria for possible bvFTD in a large longitudinal series of patients [20]. Thus, some patients with phenocopy syndrome may have a neurodegenerative pathology and a definite FTD diagnostic when a screening for the *C9orf72* mutation is applied to them. Genetic investigation for *C9orf72* must be considered in cases of suspected phFTD. Taking into account that *R406W MAPT* mutation has also been associated with slowly progressive behavioral disorder [34], the genetic screening for this mutation must also be considered in selected cases.

The issue becomes more complex with the finding of the *C9orf72* expansion in psychiatric disorders like late-onset psychosis and bipolar disorder [9]. Some authors argue that phFTD actually represents late-onset psychiatric disorders, including late-onset schizophrenia and bipolar disorder, and/or autism spectrum disorders and personality traits decompensated in old age [4, 5, 21, 31]. Indeed, there is evidence of higher frequency of psychiatric or psychological syndromes in phFTD in comparison to bvFTD [17].

The predominance of men among phFTD patients led some authors [5, 8, 10, 20, 21, 27] to hypothesize that phFTD could be a late outcome of Asperger’s disorder, which is more frequent in male than female [46, 47]. Neurodevelopmental disorders are generally more common in men [46, 47]. However, the clinical characteristics of autism spectrum disorder are evident from early childhood [31, 47, 48]. Interestingly, Gossink et al. found only one case of autism spectrum disorder among 33 patients with phFTD [17]. Late-onset schizophrenia is more common in women and its course is characterized by psychotic symptoms with relative preservation of affect.

The end-stage of bipolar disorder was proposed to be the underlying cause of phFTD [14]. In this context, it is important to discuss the “neuroprogression phenomenon” [49] to explain long-term outcomes in brain structure, cognition, functionality, and response to treatments in patients with bipolar disorder. The neuroprogression model, defined by the pathological reorganization and changes of the central nervous system along the course of severe mental disorders, would occur due to successive insults, involving inflammation and oxidative stress, directly related to repeated acute episodes. This theoretical model may include the diagnosis of phFTD syndrome as a possible outcome for the natural course of bipolar disorder and other severe psychiatric disorders.

Cluster C personality traits seem to be more frequent among phFTD patients compared to the bvFTD group [17]. It is controversial whether personality traits change in old age. The question whether phenocopy syndrome represents a late-onset primary psychiatric disorder or a slowly neurodegenerative process remains open. phFTD does not exhibit the unequivocal features of bvFTD, such as a progressive course and neuroimaging abnormalities, neither fulfilling the typical symptoms of a primary psychiatric disorder. phFTD patients may fit the criteria for “mild behavioral impairment” (MBI). MBI is defined by late-life behavioral changes, with no functional decline and no cognitive deficits [50, 51]. Importantly, MBI patients do not fulfill the criteria for psychiatric disorders. Despite the variable outcome, it has been proposed that MBI patients have a higher risk to develop FTD [52]. MBI is a very controversial construct, with an unclear neurobiological basis, and possibly refers to a heterogeneous group of clinical conditions.

The nomenclature “phenocopy syndrome of bvFTD” also deserves critical considerations. The term “phenocopy” is usually employed to refer to “a non-genetically produced phenotype that mimics or resembles the genetically produced one” [13]. It has been used, for instance, to refer to patients with Huntington’s disease phenotype but who lack the typical genetic mutation [53]. Most cases of bvFTD are not monogenic as Huntington’s disease [1], and most studies on phFTD did not test for known pathogenic mutations related to FTD such as *C9orf72*. Therefore, the term “phenocopy” may not be the most appropriate one to refer to this complex presentation.

Some important caveats about the literature on phFTD must be highlighted: (1) Studies are limited by a small number of patients. (2) Most studies were carried out at few research centers, and it is highly possible that the same patients were enrolled in different studies from the same research group. (3) The length of follow-up is variable, with some studies including phFTD patients with a clinical follow-up as short as 1 year. It is possible that a longer follow-up would detect patients with clinical progression or imaging changes. (4) Most studies did not perform genetic investigation for *C9orf72* expansion. (5) The dearth of neuropathological data must be noticed as well.

## Conclusions

In conclusion, phFTD represents a clinical condition with the same behavioral features of typical bvFTD, but without neuroimaging abnormalities and no functional decline. Whether these cases belong to the FTD spectrum is still controversial. The next advances on biomarkers and molecular neuroimaging may provide valuable tools for the diagnosis and follow-up of these patients and also clarify the pathophysiological pathways underlying phFTD and bvFTD.

## Abbreviations

ACE-R: Addenbrooke’s Cognitive Examination-Revised
ADL: Activities of daily living
AMPS: Assessment of Motor and Process Skills
bvFTD: Behavioral variant frontotemporal dementia
*C9orf72*: Chromosome 9 open reading frame 72
CBI: Cambridge Behavioral Inventory
DAD: Disability Assessment of Dementia
DMN: Default mode network
FDG-PET: Fluorodeoxyglucose-positron emission tomography
FTD: Frontotemporal dementia
FTLD: Frontotemporal lobar degeneration
*FUS*: Fused-in-sarcoma
*MAPT*: Microtubule-associated protein tau
MBI: Mild behavioral impairment
MMSE: Mini-Mental Status Examination
MRI: Magnetic resonance imaging
phFTD: Phenocopy syndrome of behavioral variant of frontotemporal dementia
PRISMA: Preferred Reporting Items for Systematic Reviews and Meta-Analyses
*TDP-43*: *TAR DNA-binding protein 43*
WM: White matter
